# How the Morphology of Nafion-Based Membranes Affects Proton Transport †

**DOI:** 10.3390/polym13030359

**Published:** 2021-01-22

**Authors:** Ernestino Lufrano, Cataldo Simari, Maria Luisa Di Vona, Isabella Nicotera, Riccardo Narducci

**Affiliations:** 1 Department of Chemistry and Chemical Technologies—CTC, University of Calabria, via Pietro Bucci, 87036 Arcavacata di Rende, Italy; ernestino.lufrano@unical.it (E.L.); cataldo.simari@unical.it (C.S.); 2 Department of Industrial Engineering and LIME Laboratory, University of Rome Tor Vergata, Via del Politecnico 1, 00133 Rome, Italy; divona@uniroma2.it

**Keywords:** nafion, conductivity, oriented morphology, recast, uncrystallized

## Abstract

This work represents a systematic and in-depth study of how Nafion 1100 membrane preparation procedures affect both the morphology of the polymeric film and the proton transport properties of the electrolyte. The membrane preparation procedure has non-negligible consequences on the performance of the proton-exchange membrane fuel cells (PEMFC) that operate within a wide temperature range (up to 120 °C). A comparison between commercial membranes (Nafion 117 and Nafion 212) and Nafion membranes prepared by three different procedures, namely (a) Nafion-recast, (b) Nafion uncrystallized, and (c) Nafion 117-oriented, was conducted. Electrochemical Impedance Spectroscopy (EIS) and Pulsed-field gradient nuclear magnetic resonance (PFG-NMR) investigations indicated that an anisotropic morphology could be achieved when a Nafion 117 membrane was forced to expand between two fixed and nondeformable surfaces. This anisotropy increased from ~20% in the commercial membrane up to 106% in the pressed membrane, where the ionic clusters were averagely oriented (Nafion 117-oriented) parallel to the surface, leading to a strong directionality in proton transport. Among the membranes obtained by solution-cast, which generally exhibited isotropic proton transport behavior, the Nafion uncrystallized membrane showed the lowest water diffusion coefficients and conductivities, highlighting the correlation between low crystallinity and a more branched and tortuous structure of hydrophilic channels. Finally, the dynamic mechanical analysis (DMA) tests demonstrated the poor elastic modulus for both uncrystallized and oriented membranes, which should be avoided in high-temperature fuel cells.

## 1. Introduction

In recent years, researchers have taken an interest in the development of more sustainable energies, both from an economic and environmental point of view. Among the different types of fuel cells, low-medium temperature, proton-exchange membrane fuel cells (PEMFCs) are promising for the replacement of classic heat engines, especially in motor vehicles [1,2]. Among the most studied and promising materials are perfluorosulfonic acid membranes (PFSA), such as long side chain (LSC) Nafion, which has, until now, been the most widely investigated ionomer, and the more recent short side chain (SSC) Aquivion from Solvay [3,4]. PFSA are characterized by high proton conductivity and chemical inertness; the latter is due to the presence of fluorine. However, sometimes the mechanical and thermal stability are not enough for the present needs in automotive applications [5,6]. In particular, when relative humidity (RH)-temperature conditions overcome certain critical values, (70–130 °C and 95–100% RH [7]), the membranes undergo some irreversible processes that induce a decrease in their through-plane proton conductivity [8]. These phenomena are due to modifications in the bulk-transport properties, and may be observed when a membrane is constrained between the electrodes and forced to swell in a plane direction [9]. A lamellar platelet was first proposed by Fujimura et al. [10] in 1981. In 1982, Starkweather [11] proposed a layered morphology. A lamellar structure was described by Litt et al. [12] in 1997, in which ionic domains were formed out of hydrophilic layers separated by thin lamellar polytetrafluoroethylene (PTFE) crystallites. A lamellar structure was suggested by Haubold et al. [13] in 2001, and recently by Kreuer et al. [14]. Ribbon morphologies were proposed by Gebel [15] in 2000, Rubatat et al. [16], Perrin et al. [17], and more recently, in 2007, by Termonia [18]. In 2013, Alberti and coworkers prepared a large batch of these low-conducting ionomers and hypothesized a layered structure; a change from randomly-oriented to plane-oriented morphologies was proposed to explain the experimental results of low conductivity and low density [19]. The results were interpretated using the INCA method (ionomer n_c_ analysis) proposed in 2008, and later by Alberti and collaborators [5,20]. This method consists of the elaboration of n_c_/T plots, where n_c_ is the counterpressure index, that describe the counter pressure force of the ionomer matrix that balances the inner osmotic pressure of the proton solution inside the membrane at equilibrium. The use of the n_c_/T plot makes it possible to determine the history of the membranes, since the value itself and the trend slope are characteristic of every treatment that the ionomer has undergone: e.g., at 50 °C, the oriented Nafion has a n_c_ value of approximately two units, much lower than the commercial Nafion 117 membrane, which has a value of about nine units [21,22]. In 2017, some studies, using the Ionomer n_c_ Analysis (INCA) method, performed on uncrystallized Nafion, with an EW = 1100, and semicrystalline Nafion, with an EW = 1000, evidenced the relation between n_c_ and the glass transition temperature of the former and the temperature of melting of crystallites in the latter [23]. Using wide-angle (WAXD) and small angle X-ray scattering (SAXS), Moore and Martin [24] found that the as-received and solution-processed films were semicrystalline, with similar degrees of crystallinity, while the recast films were amorphous. The high-solubility and “mud-cracked” character of the recast material suggested that the colloidal morphology observed in the solution remained intact in the recast state, with little chain entanglement or coalescence between particles [1]. As demonstrated by Gebel et al., with WAXS and SAXS in 1987 [25], the polymers threated with a high boiling solvent at a high temperature increased the degree of crystallinity. In 2013, Alberti et al. [26], and in 2018, Narducci et al. [22], reported a thermal treatment assisted by DMSO to increase crystallinity, and proposed a method to avoid or limit low-conductivity phase formation. This method was extended in 2019 by Giancola et al. [4] to Aquivion. A well-defined annealing temperature (*T_ann_*), between the glass transition and the melting temperature of the crystalline phase, was chosen for the treatments performed at different times in order to increase the mechanical stability [27].

In order to further confirm and clarify the previous studies carried out using the INCA method, in this study, we analyzed the proton transport behavior by Electrochemical Impedance Spectroscopy (EIS) measurements and Pulse-Field Gradient NMR spectroscopy to obtain direct measurements of the self-diffusion coefficients. Several Nafion-based membranes were investigated and compared: (i) recast Nafion, prepared by a casting procedure from the commercial solution by using DMF as a solvent, (ii) industrial recasting Nafion *NR212*, (iii) oriented Nafion 117, obtained with a special device that mimics the structure of the fuel cell, and (iv) uncrystallized Nafion, obtained by simple evaporation of a commercial solution. All these ionomers were characterized by the same EW = 1100. In particular, our attention was focused on the oriented material, in which through-plane conductivity decay occurred due to the transformation from “random” to “oriented” ribbon-type morphologies, with semicrystalline and amorphous layers being mostly parallel to the surface of the material.

The mechanical properties of such membranes have been investigated by Dynamic Mechanical Analysis (DMA). Particular attention was paid to membranes with low or no crystallinity, i.e., those positioned in the lower part of the n_c_/T plot, where the materials were mechanically less stable, and therefore, were similar or inferior to as-received or semicrystalline materials, such as Nafion 117. Once this systematic comparison had been made, we could suggest which materials could be used in fuel cells, and which should be avoided, both for use and formation under operating conditions.

## 2. Materials and Methods 

### 2.1. Chemicals

Nafion resin solution, 5 wt% solution in a mixture of lower aliphatic alcohols and water (EW, 1100 g eq^−1^), Nafion 117 membranes (EW, 1100 g eq^−1^, 180 μm thickness), and other reagents were supplied by Sigma Aldrich (Milan, Italy) and used as received. Nafion NR212 membrane (EW 1100 g eq^−1^, 51 μm thickness) were supplied by Ion Power Inc. (New Castle, DE, USA) and subjected to thermal and chemical activation before the characterization.

### 2.2. Preparation of Nafion Uncrystallized

Nafion resin solution was cast on a Petri dish (5 mL), evaporated in air for 24 h at RT, and then placed in an oven for 15 min at 80 °C to eliminate the solvent. The resulting membranes were peeled off the Petri dish and stored in P_2_O_5_ at RT [23].

### 2.3. Preparation of Nafion 117 Oriented

**Figure 1:**
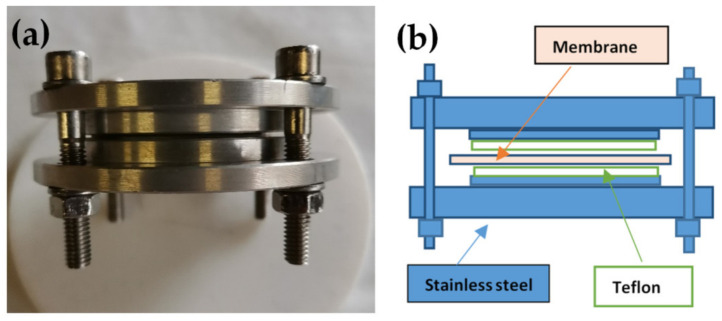
(**a**) Photo and (**b**) schematic representation of the device used for the preparation of Nafion 117 oriented.

Nafion 117 membranes were placed in the apparatus previously described in [19] (Figure 1) with a metallic disc internal diameter of 7 cm. To avoid direct contact with the metal, the plaques were covered with a Teflon foil. The whole apparatus with tightened membranes was placed inside an autoclave at 120 °C, and in liquid water for about 48 h. The pressure in the autoclave simply arose from the vapor pressure of water at 120 °C (i.e., about 2 bar). In this conformation, the membrane swelling perpendicular to the surface was not allowed, and the parallel one was facilitated with the consequent transition to oriented materials. Then, the apparatus was cooled at RT and the samples were maintained in P_2_O_5_ at RT.

### 2.4. Preparation of Nafion Recast

A Nafion recast membrane was fabricated through a typical solvent casting method. Briefly, 1 g of commercial Nafion perfluorinated resin solution was heated at about 60 °C until the complete evaporation of the solvents (water, alcohol, etc.), and then redissolved in 10 mL of Dimethylformamide (DMF) until a clear solution was obtained. Thereafter, the solution was cast on a Petri dish and placed in the oven at about 60 °C until it was dry. The membrane was finally subjected to thermal and chemical activations according to a standard method [28]. Briefly, for the membrane reinforcement, it was sandwiched and pressed between two Teflon plates and then placed in an oven at 155 °C for 15 min. Thereafter, the membrane was acid-activated by treating it with: (1) 1M HNO_3_ solution at 90 °C for 1 h to oxidize the organic impurities, (2) H_2_O_2_ (3 vol%) at 60 °C for 1 h to remove all the organic impurities, (3) 1M H_2_SO_4_ at 80 °C for 1 h to remove any metallic impurities, (4) 0.001 M ethylenediaminetetraacetic acid (EDTA) solution at RT for 1 day to remove all the paramagnetic contaminants (such as the copper contained in the Nafion commercial solution), (5) 2 M HCl at 80 ° C for 2 h, and (6) EDTA. After each acidic treatment, the membrane was rinsed three times in boiling deionized H_2_O for 10 min to remove any trace of the acids. The thickness of the dry membranes was about 50 µm.

### 2.5. Water Uptake (WU), λ and n_c_

The samples were kept in liquid water at 25 °C for 24 h in a hermetically-sealed Teflon vessel. The membranes were carefully wiped off and the mass was determined (m_wet_). They were then dried over P_2_O_5_ for 3 days and weighed (m_dry_). The WU was calculated according to Equation (1):

(1) $$\mathrm{WU}\;=\;\left(\frac{m_{\mathrm{wet}}-m_{\mathrm{dry}}}{m_{\mathrm{dry}}}\right)\times 100$$

The hydration number λ, i.e., the number of water molecules per SO_3_H group, was calculated by Equation (2):

(2) $$\lambda =\left(\frac{\mathrm{WU}}{\mathrm{IEC}\times M\left(H_{2}O\right)}\right)\times 10$$

where the ion exchange capacity (IEC) of Nafion 1100 is 0.909 meq. g^−1^ and M is the molar mass of water. The uncertainty is estimated to be about ± 0.5.

The λ values were converted into n_c_ values by the Equation (3):

(3) $$n_{c}\;=\;\frac{100}{\lambda -6}$$

This equation is valid for λ ≥ 10 [5,20].

### 2.6. ^1^H NMR Spectroscopy NMR

The NMR measurements were performed with a Bruker AVANCE 300 Wide Bore NMR spectrometer working at 300 MHz on ^1^H, and equipped with a Diff30 Z-diffusion 30 G/cm/A multinuclear probe with exchangeable RF inserts (Bruker, Milan, Italy). The self-diffusion coefficients (D) of water were determined by pulsed field gradient stimulated-echo (PFG-STE) technique [29]. The sequence consists of three 90° rf pulses (π/2-τ_1_-π/2-τ_m_-π/2) and two gradient pulses applied after the first and the third RF pulse. At time 2τ_1_ + τ_m_, the echo was found. The FT echo decays were analyzed by means of the relevant Stejskal–Tanner expression Equation (4):

(4) $$I=I_{0}{\;e}^{-\beta D}$$

where I and I_0_ represent the intensity/area of a selected resonance peak with and without gradients, respectively, D the self-diffusion coefficient, and β the field gradient parameter. Following the usual notation, the magnetic field pulses had amplitude g, duration d, and time delay Δ. Accordingly, the field gradient parameter can be defined by Equation (5):

(5) $$\beta =\left[{\left(\gamma g\delta \right)}^{2}\;\left(\Delta -\frac{\delta }{3}\right)\right]$$

The used experimental parameters were: δ = 0.8 ms, time delay ∆ = 8 ms, and the gradient amplitude varied from 100 to 900 G cm^−1^. Based on the very low standard deviation of the fitting curve and repeatability of the measurements, the uncertainties of the self-diffusion measurements were approximately 3%. The NMR samples were prepared according to the procedure described in detail elsewhere [30]. The self-diffusion coefficients were measured in the temperature range of 20 °C to 130 °C, measured every 20 °C, leaving the sample to equilibrate at each temperature for approximately 20 min.

### 2.7. Dynamic Mechanical Analysis DMA

Dynamic Mechanical Analysis (DMA) was conducted by Metravib DMA/25 analyzer equipped with a shear jaw for films clamping (Limonest, France). A dynamic stress of amplitude of 10^−4^ at 1 Hz is applied on a rectangular shaped sample (width = 3 cm; height = 1 cm), in the temperature range of 25–200 °C, with a heating scan rate of 2 °C min^−1^.

### 2.8. Electrochemical Impedance Spectroscopy (EIS)

A commercial four-electrode cell (BT-112, Scribner Associates Inc., Southern Pines, NC, USA) was adopted to measure the in-plane proton conductivity of the various Nafion membranes [31]. In this case, the membranes were cut into rectangular shapes of 25 mm × 10 mm. For the through-plane conductivity, the membrane was sandwiched between two disks of conductive carbon papers (*d* = 10.5 mm) and placed in a homemade two-electrode cell. Impedance spectra were recorded on a PGSTAT30 potentiostat/galvanostat/FRA (Metrohm Autolab B.V., Utrecht, The Netherlands) at OCV, over a frequency range between 1 Hz to 1 MHz, with an oscillating potential of about 10 mV. The resulting impedance data were analyzed by Metrohm Autolab NOVA software. From the Nyquist plot, the electrolyte resistance (*R*) was extracted as the high-frequency intercept on the real axis, and the ionic conductivity (σ) was calculated according to Equation (6) and reported as an average of three independent measurements:

(6) $$\sigma =\frac{L}{RA}$$

where *L* is the distance between the electrodes and *A* is the active area.

The in-plane and through-plane proton conductivities were measured as a function of the temperature, in the range of 20–120 °C, at 90% RH, leaving the sample to equilibrate for at least 30 min before each measurement. A humidification system (Fuel Cells Technologies, Inc Albuquerque, NM, USA) directly connected to the cell was used to finely control temperature and RH.

## 3. Results and Discussion

### 3.1. Conductivity Study (Through-Plane vs. In-Plane)

**Figure 2:**
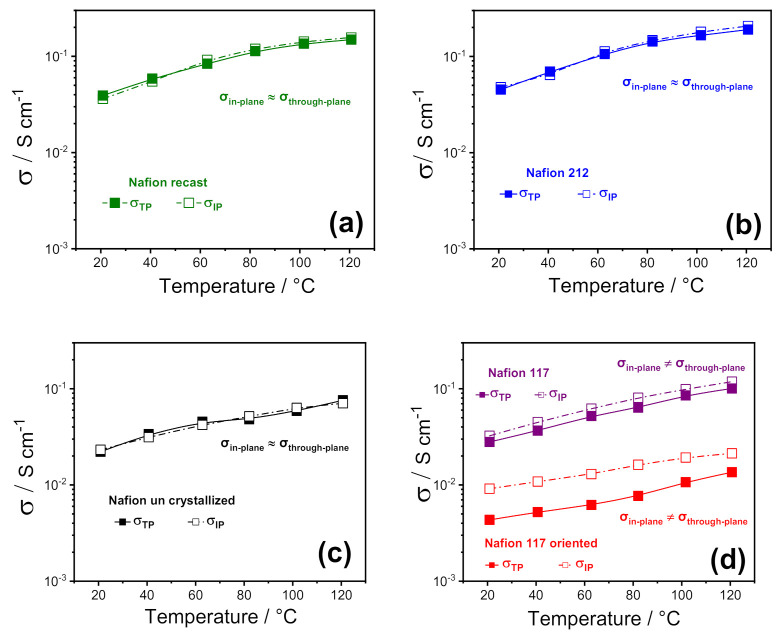
In-plane vs. through-plane proton conductivities of (**a**) Nafion recast, (**b**) Nafion 212; (**c**) Nafion un crystallized and (**d**) Nafion 117 as received and Nafion 117 oriented membranes as a function of temperature (20–120 °C) at 90% RH%. (The error bars are not reported because they are smaller than the size of the symbols).

To highlight any sort of anisotropy in the membrane morphology induced by the fabrication procedure, the various Nafion 1100 membranes were investigated by electrochemical impedance spectroscopy (EIS) by using two cell configurations, through-plane (σ_TP_) and in-plane (σ_IP_). Figure 2 illustrates the comparison between through-plane and in-plane proton conductivity of the PFSA membranes, in the temperature range of 20 °C to 120 °C at 90% RH. Furthermore, for the sake of comparison among the different Nafion-based membranes, some representative values are also reported in Table 1. In 2008, Holdcroft et al. [32] first compared the in-plane and through-plane conductivity of several Nafion membranes, demonstrating that the conductivity was clearly anisotropic in extruded samples, with the σ_IP_ higher than σ_TP_, whereas it was isotropic in recast films. Furthermore, a strong correlation between the membrane thickness and the anisotropic degree was also observed: the discrepancy between in-plane and through-plane conductivity was lower for thicker membranes (i.e., 18% for Nafion 117) and increased for thinner membranes, such as Nafion 112, reaching a value of 32%.

Our findings are consistent with the outcome described by Holdcroft et al., as all the solution-cast membranes (Nafion recast, 212, and uncrystallized) exhibited isotropic conductivity, confirming the absence of any particular orientation in their polymer chains and/or in their ionic clusters. At the same time, a relatively small anisotropy was observed in the case of Nafion 117, with the conductivity parallel to the extruding direction (σ_IP_) ~ 20% higher than the through-plane. It is worth noting that the in-plane to through-plane anisotropy increased to almost 106% when Nafion 117 oriented. This suggests that the ion-conducting clusters are mostly oriented along the plane of the membrane’s surface, inducing a strong directionality to the proton transport. This is expected to greatly affect the performance of the electrolyte in the PEMFC, where the σ_TP_ is crucial. In particular, while the through-plane conductivity of Nafion 212, recast, 117, and partially uncrystallized membranes still match the requirements for PEMFC application, the low σ_TP_ of Nafion 117 oriented makes it unsuitable for practical application; for this reason, its formation in the fuel cell itself should also be avoided. Such a huge decrease in the through-plane conductivity of Nafion 117 oriented is also accompanied by an appreciable decrease of the ionomer density. It is found to be 1.7–1.8 g cm^−3^, a little higher than that found in the previous work, i.e., 1.4–1.5 g cm^−3^, probably due to some small differences in the procedure, and because the previous device had a smaller diameter than the one used here. However, such a density reduction is sufficient to cause the drop in conductivity, that we attribute to the transition of ribbon phases already present in commercial Nafion, from a random orientation to parallel direction of the surface, probably causing less/worsened interconnection between the ion channels, giving rise to lower through-plane conductivity.

### 3.2. Hydration Number (λ) and Counterpressure Index (n_c_) 

From the water uptake measured for each membrane and reported in Table 2, it was possible to calculate λ and n_c_. Based on these values, we can determine, without building the whole n_c_/T plots at different temperatures, the position in the plot at 25 °C of the single sample, suggesting the type of Nafion we are investigating and what kind of treatment it has previously undergone.

**Figure 3:**
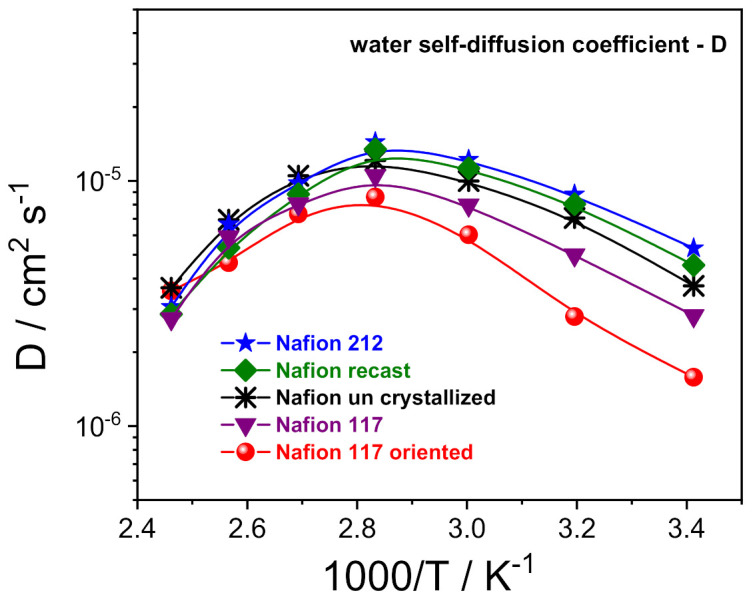
Self-diffusion coefficients as a function of the temperature (from 20 °C to 130 °C) of the water confined in the various PFSA membranes.

The values of n_c_ fall with good approximation in Figure 3 of the reference 17, suggesting the good reproducibility of the system [19].

From Table 2, Nafion 212 has the lowest water uptake and the highest value of n_c_. One need to consider that, according to Alberti et al. [5], one n_c_ unit is equivalent to an increase of Young’s modulus by about 6.5 MPa, with the higher the value having the greater mechanical resistance.

The highest value of WU was reached for the uncrystallized membrane. The thickness of this sample is comparable to commercial Nafion 212, i.e., about 50 µm. Both membranes have been obtained by recasting method; however, the preparation of the uncrystallized does not use a high-boiling solvent, in contrast to Nafion 212. In fact, as reported in the literature (e.g., Alberti [26], Gebel [25], Moore [24]), the use of high-boiling solvents such as DMF and DMSO increases the crystallinity on both preformed and casting membranes. Therefore, the uncrystallized Nafion membrane demonstrated a weaker structure. Finally, the Nafion 117 oriented presented a lower density, had a higher equivalent volume, and therefore, had a greater willingness to host water [19].

#### H PFG NMR Investigation

^1^H-PFG NMR experiments allowed us to investigate the molecular dynamics of the water confined inside the prepared electrolytes, via the direct measurements of the water self-diffusion coefficients (D) [33,34]. Figure 3 shows the Arrhenius plot of the water self-diffusion coefficients measured on completely swelled membranes in the temperature range between 20 and 130 °C. For all the membranes, the diffusivity increases with temperature up to 80 °C due to thermal energy, then drops precipitously as a consequence of the evaporation of the bulk-like water fraction. In this regard, once above 80 °C, the D values of the various Nafion membranes become quite comparable and merge towards the same value. This is indicative that the “bound-water” fraction that remains in the ion clusters as hydration to sulfonic groups is almost invariable, regardless of the casting procedure/preparation of the polymeric film. Instead, the analysis of D in the temperature range of 20–80 °C allows us to clarify the relationship between proton transport properties and a membrane’s nanomorphology:

Despite the highest water uptake (29 wt%), uncrystallized Nafion displays slightly lower D than both Nafion 212 and Nafion recast (*w.u.* 22 wt% and 24 wt%, respectively). Likely, the decreasing of the crystallinity degree induces a more branched channel structuring, increasing the diffusion path’s tortuosity for water, thus slowing the overall diffusivity.

A Nafion membrane structure more regular with a lamellar morphology of the polymer chains reduces the mobility of water molecules, being limited to only two directions. Indeed, Nafion 117 and, even more, the Nafion 117 oriented, show a significant decrease of water diffusion.

### 3.3. Dynamic Mechanical Analysis

**Figure 4:**
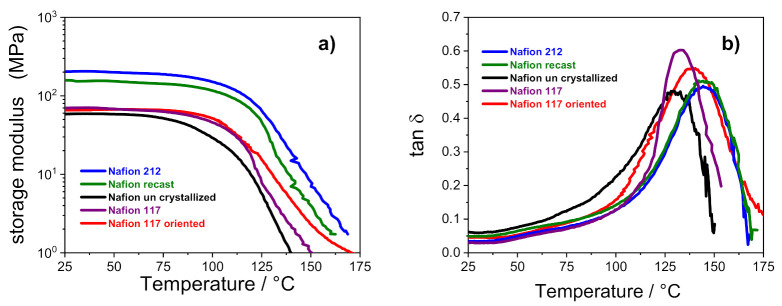
Storage modulus (**a**) and tan δ (**b**) versus temperature of PFSA membranes.

Figure 4 shows the temperature evolution of storage modulus (E’) and dumping factor (tan δ) of all the membranes. It can be observed that the storage moduli of Nafion 212 and Nafion recast (both thermally activated before the characterization) are almost comparable, and are the highest among the investigated membranes: E’ for these membranes is ca. 200 MPa, which is almost 3.5-fold higher than the other samples in which the storage modulus is ca. 60 MPa. 

This outcome confirms that the thermal activation procedure is crucial to achieving tough and mechanically stable polymeric film. As noted in Table 2, the Nafion 212 and recast PEMs show a high value of n_c_, which, according to Alberti et al. [5], is related to a greater mechanical stability. It is worth noting that the storage modulus of uncrystallized Nafion starts decreasing at 70 °C, whereas the other membranes show a high E’ until 100 °C. As described in the experimental methods, the Nafion uncrystallized was prepared in absence of high-boiling solvents. This reduces the overall crystallinity of the membrane and clearly has a detrimental effect on its thermal resistance. The dumping factor plots showed in Figure 4b further elucidates the relationship between thermal resistance and membranes architecture. The tan δ profile of each Nafion membrane is characterized by a single peak in the high temperature region that is typically related to the α transition (*T*_g_) of the ionic clusters [35,36]. In the case of solution-cast membranes, the *T*_g_ of uncrystallized Nafion is shifted to lower temperatures (i.e., 128 °C) in comparison with Nafion 212 and Nafion recast, which show the α transition at ~145 °C. The outcome, which is clearly amenable to the lower crystallinity of the uncrystallized Nafion, de facto limits the practical application of this PEM in high-temperature fuel cell devices, which has a temperature target fixed by DOE of 120 °C. Turning our attention to the extruded membranes, the higher *T*_g_ of Nafion 117 oriented compared to the parental Nafion 117 (i.e., 139 °C vs. 132 °C, respectively) is compatible with a reduced flexibility of the polymer chains after strict orientation of its ionic clusters, which need more energy to move, leading to a higher relaxation temperature.

## 4. Conclusions

This study extends our knowledge of the morphology of Nafion membranes from previous INCA method results. In particular, it has been argued that the decay of the through-plane conductivity in Nafion-oriented membranes is the result of the formation of “oriented-ribbon”-type morphologies and poor connections between ion clusters, which macroscopically is accompanied by a consistent decrease in density. In this study, both in-plane and through-plane conductivities were investigated using a wide range of temperatures (20–120 °C). There was evidence of a strong anisotropy between σ_TP_ and σ_IP_ in the case of extruded membranes, which increases from 20% for the Nafion 117 commercial membrane to almost 106% in the Nafion 117-oriented membrane. In this last sample, the lamellar-like morphology significantly reduced the water diffusivity because it was restricted in only two directions.

Regarding the uncrystallized Nafion, in previous studies, we observed a strong lowering of n_c_/T plots due to either an important decreasing in crystallinity, or more likely due to a decomposition of the hydrogen bonds between adjacent ribbons. The high water uptake ability of this membrane, however, is not accompanied by a corresponding high conductivity. On the contrary, both the lower proton conductivity and water diffusion, compared to Nafion 212 and Nafion recast, suggest that the decreased degree of crystallinity induces more branched channel structuring, increasing tortuosity and slowing down the overall transport properties. In addition, the β-transition shifts at 20 °C lower than the other solution-cast membranes, which, along with having a lower storage modulus, definitely limits the practical application of uncrystallized Nafion. On the other hand, materials with a high degree of crystallinity induced by the use of high-boiling solvents, such as DMF and DMSO, have optimal structural characteristics for use in medium/high-temperature fuel cells.

## Figures and Tables

**Table 1:** Proton conductivity [mS cm^−1^], @ RH 90% and for three temperature values, of the PFSA membranes (along with the standard deviation resulting from three independent measurements).

Membranes	40 °C		80 °C		120 °C	
	In-Plane	Through-Plane	In-Plane	Through-Plane	In-Plane	Through-Plane
Nafion 117	40.0 ± 0.8	36.7 ± 1.2	76.5 ± 1.7	63.6 ± 1.3	118.4 ± 1.7	100.6 ± 1.6
Nafion 212	74.1 ± 1.5	76.1 ± 1.6	157.0 ± 2.1	153.1 ± 2.1	220.0 ± 2.1	213.1 ± 2.2
Nafion recast	54.8 ± 1.2	58.7 ± 1.3	119.7 ± 1.9	113.3 ± 1.8	151.0 ± 2.0	149.1 ± 1.9
Nafion uncrystallized	31.2 ± 1.1	30 ± 0.9	52.1 ± 1.1	56.1 ± 1.4	71.3 ± 1.3	76.1 ± 1.6
Nafion 117 oriented	11.07 ± 0.6	5.1 ± 0.3	16.0 ± 0.8	8.08 ± 0.3	21.0 ± 0.8	14.09 ± 0.7

**Table 2:** Thickness, WU%, lambda, and nc for Nafion 117, NR212, “recast”, “uncrystallized” and “oriented” at 25 °C in liquid water for 24 h.

Membranes	Thickness [μm]	WU [wt%]	λ	n_c_
Nafion 117	180 ± 6	24	14.6	11.6
Nafion 212	51 ± 2	22	13.4	13.5
Nafion recast	50 ± 1	24	14.6	11.6
Nafion uncrystallized	59 ± 2	29	17.7	8.6
Nafion 117 oriented	160 ± 5	25	15.3	10.8
